# Identification of Novel *Acinetobacter baumannii* Host Fatty Acid Stress Adaptation Strategies

**DOI:** 10.1128/mBio.02056-18

**Published:** 2019-02-05

**Authors:** Jhih-Hang Jiang, Karl A. Hassan, Stephanie L. Begg, Thusitha W. T. Rupasinghe, Varsha Naidu, Victoria G. Pederick, Marjan Khorvash, Jonathan J. Whittall, James C. Paton, Ian T. Paulsen, Christopher A. McDevitt, Anton Y. Peleg, Bart A. Eijkelkamp

**Affiliations:** a Infection and Immunity Program, Monash Biomedicine Discovery Institute and Department of Microbiology, Monash University, Clayton, Victoria, Australia; b School of Environmental and Life Sciences, University of Newcastle, Callaghan, New South Wales, Australia; c Research Centre for Infectious Diseases, School of Biological Sciences, University of Adelaide, Adelaide, South Australia, Australia; d Department of Microbiology and Immunology, The Peter Doherty Institute for Infection and Immunity, University of Melbourne, Melbourne, Victoria, Australia; e Metabolomics Australia, School of BioSciences, University of Melbourne, Melbourne, Victoria, Australia; f Department of Chemistry and Biomolecular Sciences, Macquarie University, Sydney, New South Wales, Australia; g School of Pharmacy and Medical Sciences, Sansom Institute for Health Research, University of South Australia, Adelaide, South Australia, Australia; h Department of Infectious Diseases, The Alfred Hospital and Central Clinical School, Monash University, Melbourne, Victoria, Australia; Louis Stokes Veterans Affairs Medical Center

**Keywords:** AdeIJK, antimicrobial host lipids, RND efflux, β-oxidation, free fatty acids, lipidomics

## Abstract

Free fatty acids hold important immune-modulatory roles during infection. However, the host’s long-chain polyunsaturated fatty acids, not commonly found in the membranes of bacterial pathogens, also have significant broad-spectrum antibacterial potential. Of these, the omega-6 fatty acid arachidonic acid (AA) and the omega-3 fatty acid decosahexaenoic acid (DHA) are highly abundant; hence, we investigated their effects on the multidrug-resistant human pathogen Acinetobacter baumannii. Our analyses reveal that AA and DHA incorporate into the A. baumannii bacterial membrane and impact bacterial fitness and membrane integrity, with DHA having a more pronounced effect. Through transcriptional profiling and mutant analyses, we show that the A. baumannii β-oxidation pathway plays a protective role against AA and DHA, by limiting their incorporation into the phospholipids of the bacterial membrane. Furthermore, our study identified a second bacterial membrane protection system mediated by the AdeIJK efflux system, which modulates the lipid content of the membrane via direct efflux of lipids other than AA and DHA, thereby providing a novel function for this major efflux system in A. baumannii. This is the first study to examine the antimicrobial effects of host fatty acids on A. baumannii and highlights the potential of AA and DHA to protect against A. baumannii infections.

## Abstract (precis)

A shift in the Western diet since the industrial revolution has resulted in a dramatic increase in the consumption of omega-6 fatty acids, with a concurrent decrease in the consumption of omega-3 fatty acids. This decrease in omega-3 fatty acid consumption has been associated with significant disease burden, including increased susceptibility to infectious diseases. Here we provide evidence that DHA, an omega-3 fatty acid, has superior antimicrobial effects upon the highly drug-resistant pathogen Acinetobacter baumannii, thereby providing insights into one of the potential health benefits of omega-3 fatty acids. The identification and characterization of two novel bacterial membrane protective mechanisms against host fatty acids provide important insights into A. baumannii adaptation during disease. Furthermore, we describe a novel role for the major multidrug efflux system AdeIJK in A. baumannii membrane maintenance and lipid transport. This core function, beyond drug efflux, increases the appeal of AdeIJK as a therapeutic target.

## OBSERVATION

### Omega-3 and omega-6 fatty acids have distinct anti-*Acinetobacter* potential.

**FIG 1:**
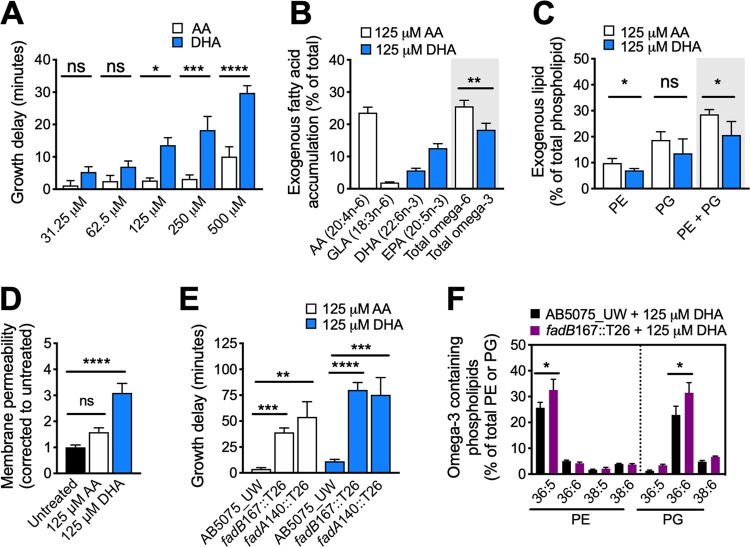
(A) The effects of long-chain polyunsaturated fatty acids (LC-PUFAs) on A. baumannii growth were determined by growing A. baumannii AB5075_UW in Luria-Bertani (LB) broth without LC-PUFA stress or with arachidonic acid (AA) or docosahexaenoic acid (DHA). The AA or DHA stress was quantified by comparing the 50% effective concentration (EC_50_), which was calculated using Prism 8 (GraphPad) from the optical density at 600 nm (OD_600_) measurements taken every 30 min, as per previous work (20). The EC_50_ is the time (minutes) when cultures have reached 50% of the maximum cell density, thereby representing mid-log growth. (B) The cell-associated omega-6 fatty acids (AA, GLA, and total n-6) and omega-3 fatty acids (DHA, EPA, and total n-3) were examined by gas chromatography following growth of strain AB5075_UW in the presence of 125 μM AA- or DHA-supplemented LB, respectively, using routine methods (19, 20). (C) The modification of exogenous fatty acids into phospholipids, phosphatidylethanolamine (PE), and phosphatidylglycerol (PG), was quantified in AB5075_UW cells using liquid chromatography-mass spectrometry (LC-MS) and underwent further species verification by tandem MS (MS/MS) following published protocols (19, 21). (D) The membrane permeability of AB5075_UW and *adeJ*121::T26 cells was examined by exposing the cells to 5 μM Sytox for 5 min, followed by extensive washing and analysis on a PHERAstar spectrophotometer at excitation 485/emission 520 (BMG Labtech). (E) The effects of 125 μM AA or DHA on A. baumannii AB5075_UW and the *fadB* and *fadA* Tn insertion mutant derivative *fadB*167::T26 and *fadA*140::T26 strains (14), respectively. The growth delay (in minutes) was calculated by comparing the EC_50_s. (F) The conversion of exogenous DHA into cell-associated PE or PG phospholipids in AB5075_UW or *fadB*167::T26 cells as determined by LC-MS. For all panels, the results are the mean ± standard error of the mean (SEM) from at least biological triplicates. Statistical analyses were performed using a one-way analysis of variance (ANOVA) (A, C, D, and E) or a Student's *t* test (B and F). ns, not significant; *, *P* < 0.05; **, *P* < 0.01; ***, *P* < 0.001; ****, *P* < 0.0001.

Considering the major disease burden associated with an imbalance in the human omega-3 and -6 fatty acid status (1, 2) and the well-documented antibacterial activity of long-chain polyunsaturated fatty acids (LC-PUFAs) (3, 4), we examined the impact of omega-3 and -6 fatty acids on Acinetobacter baumannii. A. baumannii is a human pathogen that is of primary concern in the hospital environment, where its exceptional capacity for antimicrobial resistance allows it to cause significant morbidity and mortality in susceptible patients (5, 6). Here, we investigated the effect of arachidonic acid (AA; 20:4n-6) or docosahexaenoic acid (DHA; 22:6n-3) at 31.25 to 500 µM upon the growth of A. baumannii strain AB5075_UW. These concentrations and the magnitude of variation are physiologically relevant, considering differences greater than 10-fold in serum LC-PUFAs between individuals can be seen, depending largely on dietary intake (7). We found that both LC-PUFAs induced growth perturbation, but this was more pronounced with DHA compared to AA exposure (Fig. 1A). Interestingly, preexposure to DHA did not affect the cell’s subsequent growth dynamics in the presence of DHA (see Fig. S1A in the supplemental material). Examination of alternative LC-PUFA fatty acids revealed that the antimicrobial potential of omega-3 and -6 fatty acids may be conserved because γ-linolenic acid (GLA; omega-6) and eicosapentaenoic acid (EPA; omega-3) have similar impacts on the growth of strain AB5075_UW as AA and DHA, respectively (Fig. S1B and C).

10.1128/mBio.02056-18.1 FIG S1 (A) Overnight AB5075_UW cultures were diluted to OD_600_ of 0.01 in 4 ml LB broth and grown to an OD_600_ of 0.7 with or without 125 μM DHA. Cells were diluted to an OD_600_ of 0.01, and growth with or without 125 μM DHA was examined by OD_600_ measurements taken every 30 min. The effect of preexposure to DHA (squares; pre-expo) upon subsequent DHA tolerance was examined and compared to that in untreated seeding cultures (circles). The results are the mean from at least biological triplicates. Error bars ± SEM where not visible are overlapped by the data points. (B and C) Growth of AB5075_UW cells in the presence of omega-6 (B) or omega-3 (C) fatty acids. Overnight cultures were diluted to an OD_600_ of 0.01, and growth with 125 μM AA (solid gray squares), 125 μM GLA (open gray squares), 125 μM DHA (solid blue squares), 125 μM EPA (open blue squares), or without LC-PUFA treatment (solid black circles) was examined by OD_600_ measurements taken every 30 min. (D) The effects of 125 μM AA or DHA on the endogenous fatty acids of A. baumannii AB5075_UW cells were determined by gas chromatography (GC). The results are the mean ± SEM from at least biological triplicates. Statistical analyses were performed using a one-way ANOVA (ns, not significant; ****, *P* < 0.0001). (E) Transcription levels of genes that encode the A. baumannii FASII pathway (*bioA*, *bioB*, *acpP*, *accA*, *fabG*, *fabH*, *fabI*, and *fabZ*) and the β-oxidation pathway (β-ox; *fadB*) in AB5075_UW cells grown with or without 125 μM AA or DHA were examined by qRT-PCR, using the GAPDH gene as a reference gene, and data are presented as corrected against untreated cells. Oligonucleotide sequences can be found in Table S1. The results are the mean ± SEM from at least biological triplicates. Statistical analyses were performed using a one-sample *t* test (*, *P* < 0.05; **, *P* < 0.01). (F) The abundance of DHA and EPA in AB5075_UW or *fadB*167::T26 cells following treatment with 125 μM DHA as determined by GC. The results are the mean ± SEM from at least biological triplicates. Statistical analyses were performed using a one-way ANOVA (ns, not significant). Download FIG S1, TIF file, 0.4 MB. Copyright © 2019 Jiang et al. 2019 Jiang et al. This content is distributed under the terms of the Creative Commons Attribution 4.0 International license.

We next examined the relative accumulation of the exogenous fatty acids in A. baumannii upon *in vitro* treatment with either 125 μM AA or 125 μM DHA, representing the lowest concentration at which the omega-3 fatty acid shows a significantly greater antimicrobial potential compared to the omega-6 fatty acid (Fig. 1A; Fig S1B and C). This revealed that not only are the exogenous fatty acids incorporated, but A. baumannii partly degrades AA and DHA, generating GLA (18:3n-6) and EPA (20:5n-3), respectively (Fig. 1B). Examination of the two major phospholipid species phosphatidylethanolamine (PE) and phosphatidylglycerol (PG) suggested that the exogenous lipids were readily incorporated into phospholipids (28% and 20% of total phospholipids after AA and DHA treatment, respectively) (Fig. 1C). This strongly indicates that the bacterium expresses systems that actively interact with host fatty acids. Based on the level of host fatty acid incorporation into the bacterial membrane, we investigated the impact of AA or DHA on bacterial membrane integrity (Fig. 1D). Treatment with AA or DHA increased the membrane permeability, but this was most significant (3.1-fold) after exposure to DHA (Fig. 1D). Overall, despite AA accumulating in the bacterial cell at higher levels, the longer and more desaturated host fatty acid DHA exerted a greater effect on A. baumannii growth and membrane integrity.

Membrane phospholipids play a key role in the defense against antimicrobials, including host fatty acids (8). Hence, we examined the effect of AA or DHA treatment upon the abundance of the endogenous A. baumannii fatty acids. The results showed a highly specific depletion (≥50%) of the monounsaturated fatty acids 16:1n-7 and 18:1n-9 (Fig. S1D). This could not be accounted for through the transcriptional dysregulation of the FASII pathway, as seen in Gram-positive species (9). However, the *fadAB* operon (ABUW_3573-3572) was significantly upregulated upon treatment with AA or DHA (Fig. S1E). The *fadAB* operon encodes critical components of the fatty acid β-oxidation pathway, indicating fatty acid degradation may provide protection against LC-PUFAs. Indeed, the fitness of *fadA*- and *fadB*-inactivated mutants was significantly compromised under AA and DHA stress (Fig. 1E). Interestingly, the accumulation of DHA or EPA was not affected by mutation of *fadB*, despite its hypersusceptibility to DHA (Fig. S1F). Instead, we observed that of the PE and PG species with exogenous fatty acid incorporated, PE(36:5) and PG(36:6), the levels were significantly higher in the *fadB* mutant strain upon treatment with DHA (Fig. 1F). This suggests that the β-oxidation pathway is likely to restrict conversion of DHA and its derivatives into phospholipids. Collectively, these data indicate that the β-oxidation pathway contributes to protection against LC-PUFAs, which may impact the success of A. baumannii as a human pathogen.

### The AdeIJK RND efflux system is involved in LC-PUFA resistance.

**FIG 2:**
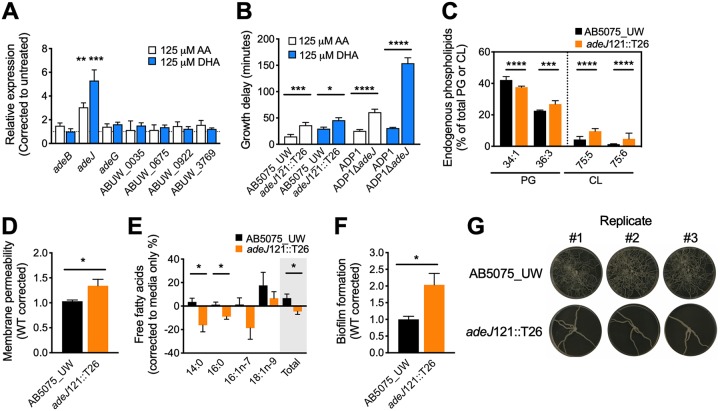
(A) Transcription levels of genes that encode putative A. baumannii resistance nodulation cell division (RND) efflux pumps in AB5075_UW cells grown in LB media with or without AA or DHA were examined by quantitative reverse transcription-PCR (qRT-PCR), using the glyceraldehyde-3-phosphate dehydrogenase (GAPDH) gene as a reference gene, and data were corrected against untreated cells as per previous analyses (19, 20). AB5075_UW cultures were grown to an optical density at 600 nm (OD_600_) of 0.5 and were exposed to 125 μM AA or DHA for 30 min prior to processing for RNA isolation. Oligonucleotide sequences can be found in Table S1 in the supplemental material. The results are the mean ± SEM from at least biological triplicates. Statistical analyses were performed using a one-sample *t* test (**, *P* < 0.01; ***, *P* < 0.001). (B) The effect of 125 μM AA or DHA on *adeJ*-inactivated A. baumannii AB5075_UW (*adeJ*121::T26) and A. baylyi ADP1 (Δ*adeJ*) mutants (14, 15). The growth delay (in minutes) was calculated by comparing the 50% effective concentration (EC_50_) (20). The results are the mean ± SEM from at least biological triplicates. Statistical analyses were performed using a one-way ANOVA (*, *P* < 0.05; ***, *P* < 0.001; ****, *P* < 0.0001). (C) Endogenous phosphatidylglycerol (PG) and cardiolipin (CL) species that were significantly affected by mutation of *adeJ* in strain AB5075_UW (*adeJ*121::T26). The results are the mean ± SEM from at least biological triplicates. Statistical analyses were performed using a two-way ANOVA on all PG or CL species identified by LC-MS, which can be found in Table S2 in the supplemental material (***, *P* < 0.001; ****, *P* < 0.0001). (D) The membrane permeability of AB5075_UW and *adeJ*121::T26 cells was examined by exposing the cells to 5 μM Sytox for 5 min, followed by extensive washing and analysis on a PHERAstar spectrophotometer at excitation 485/emission 520 (BMG Labtech). The results are the mean ± SEM from at least biological triplicates. Statistical analyses were performed using a Student's *t* test (*, *P* < 0.05). (E) The fatty acids in the media were analyzed by gas chromatography following growth of the AB5075_UW or *adeJ*121::T26 strains. The baseline represents media in which no bacteria were grown. Samples were first centrifuged at low speed (300 × *g* for 5 min), with the supernatant then passed through a 0.4 μm-pore filter and insoluble material subsequently removed by ultracentrifugation (100,000 × *g* for 1 h). The results are the mean ± SEM from at least biological triplicates. Statistical analyses were performed using a Student's *t* test (*, *P* < 0.05). (F) The ability of AB5075_UW or *adeJ*121::T26 cells to form biofilms was assessed after 24 h of static growth in a polystyrene 96-well plate. Cells were stained using 0.1% crystal violet, with the absorbance determined at 590 nm (21, 22). The results are the mean ± SEM from at least biological triplicates. Statistical analyses were performed using a Student's *t* test (*, *P* < 0.05). (G) Surface motility of AB5075_UW or *adeJ*121::T26 cells was examined by inoculating the center of a semisolid LB plate (0.25% agar) and incubating for 18 h (22). The aberrant motility phenotype was observed in all 3 independent experiments (replicates 1, 2, and 3).

The A. baumannii resistance nodulation cell division (RND) family of efflux systems has been associated with *in vivo* survival (10). Hence, we first studied their involvement in LC-PUFA stress resistance by examining the transcriptional responsiveness upon AA or DHA supplementation. Interestingly, AA and DHA induced the specific upregulation of *adeJ* (Fig. 2A). *adeJ* is part of the A. baumannii core genome and, in combination with *adeI* and *adeK*, encodes the complete RND efflux system (AdeIJK) that plays an important role in A. baumannii multidrug resistance (10, 11). In recent years, several studies have revealed the RND efflux systems of *Acinetobacter* species, including AdeIJK, to play roles in virulence and virulence-associated phenotypes (11, 12). Although their exact mode of action remains unknown, it has been postulated to involve lipid homeostasis (13). We examined the effect of *adeJ* inactivation on susceptibility to AA and DHA in both A. baumannii strain AB5075_UW (14) and Acinetobacter baylyi strain ADP1 (15). Growth delays were observed in both *adeJ* mutants (Fig. 2B), confirming that AdeIJK plays a role in protection against AA and DHA and that this function is conserved between at least two different *Acinetobacter* species. Expression of *adeIJK* is repressed by AdeN (16); hence, we analyzed the growth dynamics of an *adeN* mutation in A. baumannii. Consistent with previous reports (10), the growth rate of the *adeN* mutant is compromised compared to that of the parental strain (see Fig. S2A in the supplemental material). However, growth rates in the presence of DHA were similar between the *adeN* mutant and wild type (WT) (Fig. S2A), indicating that DHA-mediated *adeIJK* derepression provides A. baumannii with DHA resistance at a level similar to that when *adeN* has been inactivated. We then hypothesized that the AdeIJK RND efflux system was responsible for the export of the exogenous fatty acid as a protection mechanism. Hence, we examined the accumulation of DHA in the *adeJ* mutant, but found that the accumulation of DHA and its derivative EPA, as well as the conversion into phospholipids, was significantly lower in the mutant compared to the parental strain (Fig. S2B and C). We then ascertained whether AdeIJK may be involved in membrane modulation by removing endogenous fatty acids from the membrane to achieve lipid homeostasis, similar to the EmhABC RND efflux system of Pseudomonas fluorescens (17). Lipid analyses demonstrated that the concentrations of two endogenous species of PG and two of cardiolipin were affected by *adeJ* mutation prior to treatment (Fig. 2C), with eight species in total being affected posttreatment with DHA (Fig. S2D). These findings implicate a role for AdeIJK in membrane modulation. Indeed, even without treatment, AdeJ-mediated changes in the phospholipids affected the membrane integrity, as the permeability of the *adeJ* mutant was significantly greater than that of the WT (Fig. 2D). We then examined the fatty acids in the media following growth of the WT or *adeJ* mutant (Fig. 2E). Consistent with a role for AdeJ in lipid efflux, growth of the WT resulted in an increase in fatty acids in the media, whereas growth of the mutant strain resulted in fatty acid depletion (Fig. 2E). Hence, we speculate that the differences seen between the WT and *adeJ* mutant could most likely be ascribed to AdeIJK-mediated export of fatty acids—a significant observation that indicates a novel role for AdeIJK. We also found that mutation of *adeJ* leads to an increase in biofilm formation but a decrease in surface motility in A. baumannii (Fig. 2F and G). Thus, our data suggest that the exported lipids may function as a surfactant, which renders the biofilm unstable, but promotes surface migration. Here, we show for the first time, through its identification as a protective mechanism against LC-PUFAs, that the AdeIJK RND efflux system is directly involved in the maintenance of lipid homeostasis in A. baumannii. Furthermore, through its proposed role as a lipid export system, this work has significantly advanced our understanding of the molecular basis behind AdeIJK’s far-reaching impacts on A. baumannii biofilm formation and *in vivo* fitness. Furthermore, similar to the lipid-mediated sequestration of daptomycin in Staphylococcus aureus (18, 19), the export of lipids may serve as a novel mechanism by which AdeIJK provides protection against amphiphilic or hydrophobic antimicrobials.

10.1128/mBio.02056-18.2 FIG S2 (A) Growth of AB5075_UW (wild type; circles) and the *adeN* mutant (*adeN*193::T26; triangles) following supplementation with 125 μM DHA (blue symbols and dotted lines) or without (solid black symbols and solid lines). Overnight cultures were diluted to an OD_600_ of 0.01, and growth was examined by OD_600_ measurements taken every 30 min. The results are the mean ± SEM from at least biological triplicates. Error bars, where not visible are overlapped by the data points. (B) The abundance of DHA and EPA in AB5075_UW or *adeJ*121::T26 cells following treatment with 125 μM DHA was determined by GC. The results are the mean ± SEM from at least biological triplicates. Statistical analyses were performed using a one-way ANOVA (ns, not significant; **, *P* < 0.01). (C) The incorporation of exogenous fatty acids into phospholipids, phosphatidylethanolamine (PE), and phosphatidylglycerol (PG) was quantified in AB5075_UW and *adeJ*121::T26 cells using liquid chromatography-mass spectrometry (LC-MS). The results are the mean ± SEM from at least biological triplicates. Statistical analyses were performed using a one-way ANOVA (*, *P* < 0.05; ****, *P* < 0.0001). (D) Phosphatidylethanolamine, phosphatidylglycerol, and cardiolipin (CL) species that were affected by inactivation of *adeJ* prior to or post-DHA treatment compared to the wild type (AB5075_UW). The results are the mean ± SEM from at least biological triplicates. Statistical analyses were performed using a two-way ANOVA on all PE, PG, or CL species identified by LC-MS, which can be found in Table S2 (**, *P* < 0.01; ***, *P* < 0.001; ****, *P* < 0.0001). Download FIG S2, TIF file, 0.4 MB. Copyright © 2019 Jiang et al. 2019 Jiang et al. This content is distributed under the terms of the Creative Commons Attribution 4.0 International license.

10.1128/mBio.02056-18.3 TABLE S1 Oligonucleotides used in this study. Download Table S1, DOCX file, 0.01 MB. Copyright © 2019 Jiang et al. 2019 Jiang et al. This content is distributed under the terms of the Creative Commons Attribution 4.0 International license.

10.1128/mBio.02056-18.4 TABLE S2 Phospholipids identified in strain AB5075_UW and the *adeJ*121::T26 mutant with and without DHA treatment. Download Table S2, XLSX file, 0.1 MB. Copyright © 2019 Jiang et al. 2019 Jiang et al. This content is distributed under the terms of the Creative Commons Attribution 4.0 International license.
