# Supplementary figures and images for: Identification of Novel Acinetobacter baumannii Host Fatty Acid Stress Adaptation Strategies
Source: mBio. 2019 Feb 5;10(1):e02056-18. doi: 10.1128/mBio.02056-18 (PMC6428749; doi:10.1128/mBio.02056-18)

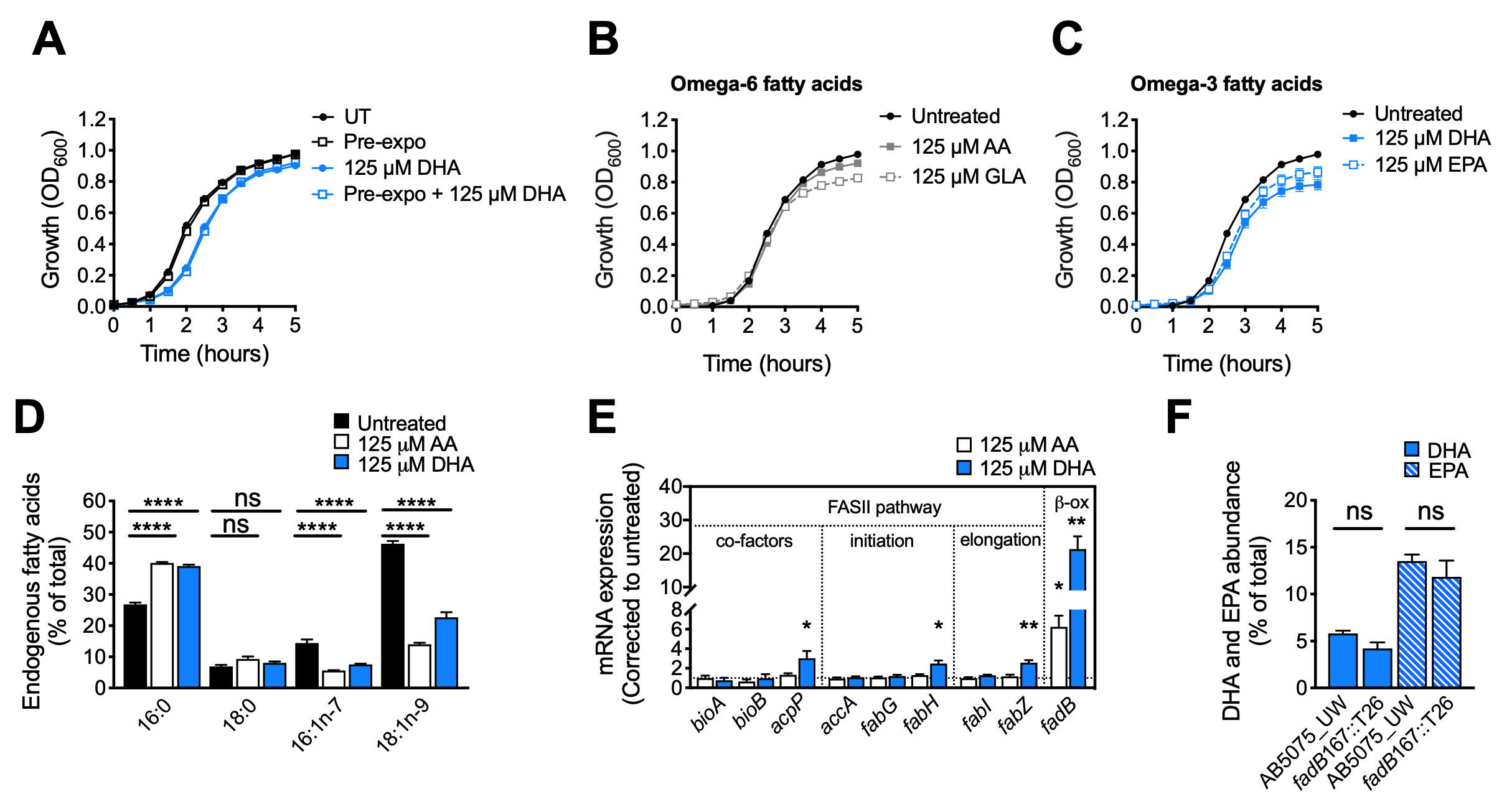

Supplement: FIG S1 [file mBio.02056-18-sf001.tif]

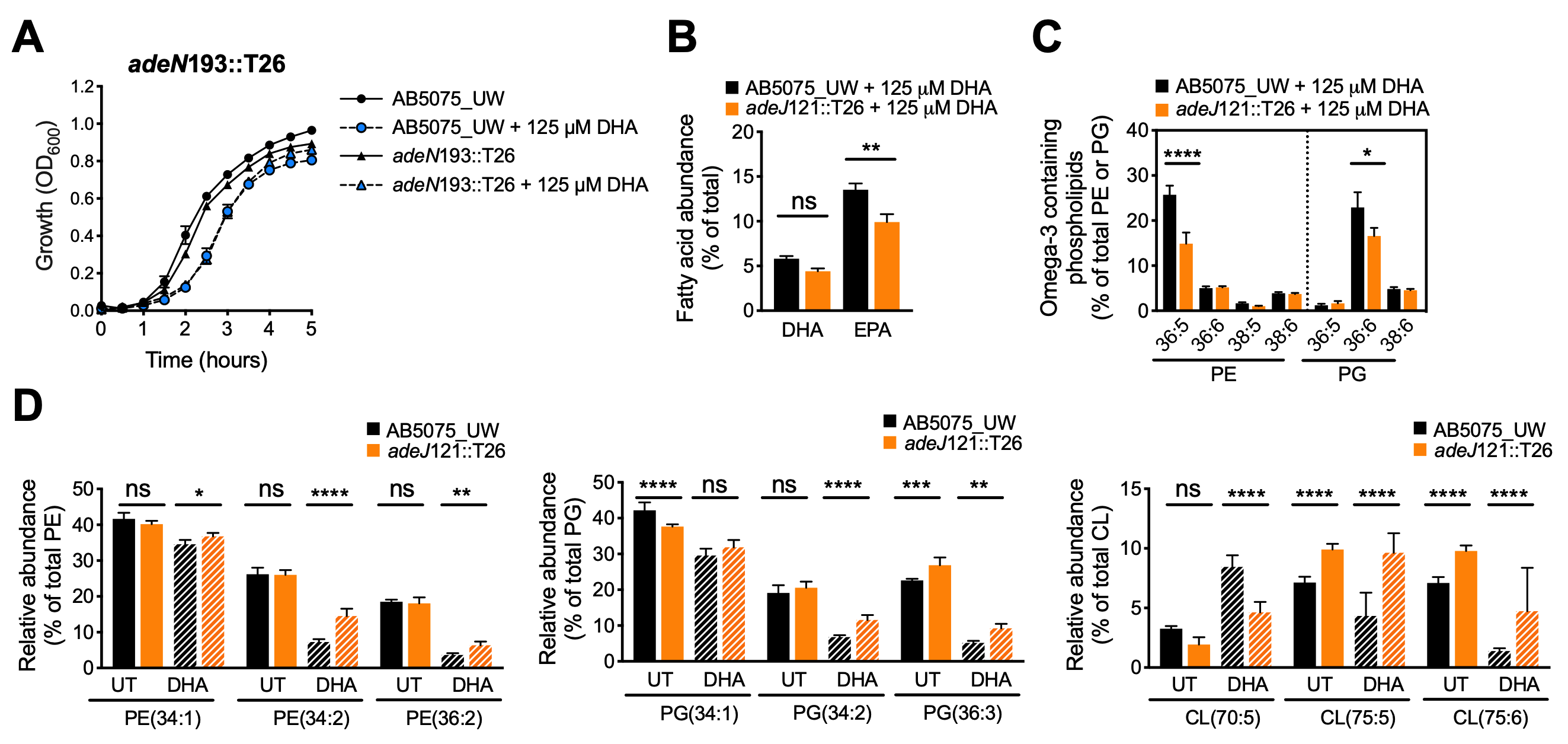

Supplement: FIG S2 [file mBio.02056-18-sf002.tif]
