# Supplementary material for: Identification of Novel Acinetobacter baumannii Host Fatty Acid Stress Adaptation Strategies
Source: mBio. 2019 Feb 5;10(1):e02056-18. doi: 10.1128/mBio.02056-18 (PMC6428749; doi:10.1128/mBio.02056-18)
Supplement: TABLE S1 [file mBio.02056-18-st001.docx]

| **Gene/locus-tag** | **Forward (5' - 3')** | **Reverse (5' - 3')** |
| --- | --- | --- |
| *accA* | TGGCTGAAGCGAAAGAAATC | ATACGGTATCATCGCCGAAC |
| *acpP* | ATATCGAACAACGCGTTAAA | CATCAGGGATAGTGATGTCGAA |
| *adeB* | GTCATGGGTTCAAGCGGTC | TTCACCCGATGACGTATCG |
| *adeG* | GCTTAAACCCAAGGGAGGC | TTGCCCTGTTAAGCCTGTC |
| *adeJ* | ATACGCAATGCGTATCTGG | ACTTGACCTTGTACAGCCG |
| *bioA* | GGTTCGGTTGCCGTTGAAGT | ATCTGATGCATGCCTGTCAC |
| *bioB* | TTTTGTATGGGTGCTGCTTG | GTTGTAATAGTCTAAACCAG |
| *fabG* | AACAAGGTGCAGGTTTGGCT | ATCCCAATCATCTTCCGACA |
| *fabH* | ATGGGGCGTTATTGCTGCAA | CGCAACGTTCATGTCGTAAG |
| *fabI* | CACGCTTGATGGTGACTTCA | GCGCTCAGAACCTTGGTAAG |
| *fabZ* | CTACAAGGGCATTTCCCAGA | TTTTAAATCTAACTCTTTCT |
| *fadB* | TCGCGGCTGTAAAAGCTAAT | CTATTGAAGATTTCGTGAAC |
| *GAPDH* | CACCGTCGTACACGTGTTGT | CAACATCACCGCCTTTTTCT |
| ABUW_0035 | CCGTCCGATCATTCTCAC | ACCACGCTGCATACAATG |
| ABUW_0675 | GCTGATGGGGCTGGTAAC | TGGGCGTAAACGGGTTTTG |
| ABUW_0922 | ATTGCCAGAGTTGAGCTAG | TGGTGGATGAGCTTAGAGG |
| ABUW_3769 | AAGCATTGGCTCAGAAGG | CAAGACAATACGGCGGTG |
